# Purpose in life promotes resilience to age-related brain burden in middle-aged adults

**DOI:** 10.1186/s13195-023-01198-6

**Published:** 2023-03-13

**Authors:** Kilian Abellaneda-Pérez, Gabriele Cattaneo, María Cabello-Toscano, Javier Solana-Sánchez, Lídia Mulet-Pons, Lídia Vaqué-Alcázar, Ruben Perellón-Alfonso, Cristina Solé-Padullés, Núria Bargalló, Josep M. Tormos, Alvaro Pascual-Leone, David Bartrés-Faz

**Affiliations:** 1 Departament de Medicina, Facultat de Medicina i Ciències de la Salut, Institut de Neurociències, Universitat de Barcelona, C/ Casanova, 143, 08036 Barcelona, Spain; 2 Institut d’Investigacions Biomèdiques August Pi i Sunyer (IDIBAPS), Barcelona, Spain; 3 Institut Guttmann, Institut Universitari de Neurorehabilitació adscrit a la UAB, Badalona, Barcelona Spain; 4 Universitat Autònoma de Barcelona, Bellaterra, Cerdanyola del Vallès, Spain; 5 Fundació Institut d’Investigació en Ciències de la Salut Germans Trias i Pujol, Badalona, Barcelona Spain; 6 Sant Pau Memory Unit, Department of Neurology, Institut d’Investigacions Biomèdiques Sant Pau-Hospital de Sant Pau, Universitat Autònoma de Barcelona, Barcelona, Spain; 7 Neuroradiology Section, Radiology Department, Diagnostic Image Center, Hospital Clinic of Barcelona, University of Barcelona, Barcelona, Spain; 8 Magnetic Resonance Image Core Facility (IDIBAPS), Barcelona, Spain; 9 Centro de Investigación Biomédica en Red de Salud Mental (CIBERSAM), Instituto de Salud Carlos III, Barcelona, Spain; 10 Centro de Investigación Traslacional San Alberto Magno, Universidad Católica de Valencia San Vicente Mártir, Valencia, Spain; 11 Hinda and Arthur Marcus Institute for Aging Research and Deanna and Sidney Wolk Center for Memory Health, Hebrew SeniorLife, Boston, MA USA; 12 Department of Neurology, Harvard Medical School, Boston, MA USA

**Keywords:** Resilience, Cognitive reserve, Brain reserve, Neuroimaging, Psychological well-being, Purpose in life, Cognition

## Abstract

**Background:**

Disease-modifying agents to counteract cognitive impairment in older age remain elusive. Hence, identifying modifiable factors promoting resilience, as the capacity of the brain to maintain cognition and function with aging and disease, is paramount. In Alzheimer’s disease (AD), education and occupation are typical cognitive reserve proxies. However, the importance of psychological factors is being increasingly recognized, as their operating biological mechanisms are elucidated. Purpose in life (PiL), one of the pillars of psychological well-being, has previously been found to reduce the deleterious effects of AD-related pathological changes on cognition. However, whether PiL operates as a resilience factor in middle-aged individuals and what are the underlying neural mechanisms remain unknown.

**Methods:**

Data was obtained from 624 middle-aged adults (mean age 53.71 ± 6.9; 303 women) from the Barcelona Brain Health Initiative cohort. Individuals with lower (LP; *N* = 146) and higher (HP; *N* = 100) PiL rates, according to the division of this variable into quintiles, were compared in terms of cognitive status, a measure reflecting brain burden (white matter lesions; WMLs), and resting-state functional connectivity, examining system segregation (SyS) parameters using 14 common brain circuits.

**Results:**

Neuropsychological status and WMLs burden did not differ between the PiL groups. However, in the LP group, greater WMLs entailed a negative impact on executive functions. Subjects in the HP group showed lower SyS of the dorsal default-mode network (dDMN), indicating lesser segregation of this network from other brain circuits. Specifically, HP individuals had greater inter-network connectivity between specific dDMN nodes, including the frontal cortex, the hippocampal formation, the midcingulate region, and the rest of the brain. Greater functional connectivity in some of these nodes positively correlated with cognitive performance.

**Conclusion:**

Expanding previous findings on AD pathology and advanced age, the present results suggest that higher rates of PiL may promote resilience against brain changes already observable in middle age. Furthermore, having a purposeful life implies larger functional integration of the dDMN, which may potentially reflect greater brain reserve associated to better cognitive function.

**Supplementary Information:**

The online version contains supplementary material available at 10.1186/s13195-023-01198-6.

## Introduction

Increased life expectancy represents one of the biggest transformations of age structure among contemporary societies [1]. This denotes an important accomplishment but also poses an enormous societal and health challenge, as advanced age is a principal risk factor for many highly prevalent and disabling disorders, including Alzheimer’s disease (AD). In this context, and despite intense research efforts and large investments from public and private sources, disease-modifying agents to counteract age-related cognitive impairment remain elusive. Given the lack of effective therapeutic interventions, identifying modifiable factors that may promote brain health and resilience along the lifespan is paramount [2–4].

According to the recent collaborative framework definitions (https://reserveandresilience.com/), resilience refers to a general term reflecting the capacity of the brain to maintain cognition and function with advancing age and disease. Within this framework, the investigation of resilience mechanisms can be approximated through the operational definitions of cognitive reserve (CR), which for experimental and observational studies recommend the inclusion of at least three main components: (1) a measure of brain change, (2) a measure of cognition, and (3) a variable that influences the relationship between 1 and 2. The latter variable or variables, which are often referred as “proxies of CR,” have classically been derived from single or composite estimations of educational attainment, occupation, or engagement in leisure activities, with overall converging evidences that higher estimations of these measures allow to counteract the impact of pathology or age-related changes on cognitive outcomes [5, 6]. More recently, other modifiable factors, such as cognitive and physical activities [7, 8], combined mental and bodily practices including meditation and yoga [9–11], sleep patterns (i.e., [12, 13]), and dietary approaches [14, 15], are also being considered as modifiable lifestyles that may promote resilience. This also includes psychological factors, which are being increasingly recognized for their contribution to brain health during the lifespan [16, 17], and as their biological substrates are clarified [18, 19]. In this context, purpose in life (PiL), included in Ryff’s prevalent model of psychological well-being [20–22], is one of the most promising psychological constructs. PiL refers to the sense that life has meaning and direction and that one’s goals and potential are being achieved or are attainable [23].

Modern conceptions of PiL stem from the philosophical writings of existential philosophy, which has its formal beginning on the dissertations published in the 1840s by Sören Kierkegaard. It is relevant to consider that, while philosophers have debated whether meaning exists and what its contingencies might be, psychologists and neuroscientists have primarily focused on the importance of experiencing meaning and purpose in one’s life, exploring how this links with human health [24]. Hence, it has been previously highlighted that having a strong sense of meaning and future-oriented goals may result in a greater capacity to tolerate challenging situations for mental and physical health [25, 26]. Moreover, recent literature has revealed that having a purposeful life is associated with the maintenance of health-promoting behaviors in advanced age [27], better use of preventive health services [28], and reduced odds of mortality [23, 29]. Furthermore, PiL relates to better cognitive and affective status [16, 30] and may delay cognitive decline and the onset of cognitive impairment [31–33], thus potentially having a role as a resilience factor against AD-related pathological changes [34]. Interestingly, PiL is a transcultural conception. In the Japanese culture, Ikigai refers to a broader concept than PiL, which has been associated with longevity and lower risk of developing functional disability and dementia [35, 36]. However, the neurobiological mechanisms underlying the beneficial effects of having a purposeful life remain poorly understood.

In this study, we aimed to investigate the relationships between PiL, common brain changes occurring in adulthood, brain functional connectivity, and cognition in a sample of healthy middle-aged individuals. More specifically, and according to the operational definition of CR described above, our first objective was to study whether PiL can influence the relationship between white matter lesions (WMLs) and cognitive status in middle-aged adults, in a similar manner as observed in pathological aging [34]. Our secondary aim was to delineate the functional brain mechanisms that could explain the expected protective effects of PiL in the face of WMLs, one of the most prevalent structural brain changes in maturity, present in almost 95% of adults over 45 years of age [37–40]. For this purpose, a measure of system segregation (SyS) was used to interrogate the dynamic neural mechanisms associated with PiL. This metric was chosen because it condenses in a single estimate of the functional connectivity of within and between brain networks’ nodes [41]. Furthermore, this measure has also been used to study brain networks across the healthy adult lifespan [42] as well as resilience in the face of AD pathology [43]. Importantly, the study of the factors and brain mechanisms providing resilience to WMLs is also of interest in the context of dementia, since these are associated with vascular and amyloid pathology in aging [44, 45], and can predict faster cognitive decline and the appearance of earlier clinical manifestations [46, 47]. Since the core components of PiL, such as the identification of personal values, are modifiable through psychological interventions (e.g., [48]), identifying the functional brain signatures through which its effects operate can provide a relevant mechanistic understanding of early interventions to enhance brain health and may even prevent dementia.

## Methods

### Participants

Data was obtained from 624 middle-aged adults (mean age 53.71 ± 6.9 years; age range: 42–67; 303 women; mean years of education [YoE] 17.1 ± 3.8 years, YoE range: 8–34) from the Barcelona Brain Health Initiative (BBHI; https://bbhi.cat/en/). This is an ongoing longitudinal cohort study investigating the determinants of brain and mental health in middle-aged individuals [3, 49]. For the present work, participants were included if they met the following criteria: (i) completed a PiL questionnaire (see below) and a neuropsychological assessment and (ii) T1- and T2-weighted and resting-state functional magnetic resonance imaging (rs-fMRI) scans were available. Participants were excluded if they (i) had any neurological or psychiatric diagnosis, (ii) had below normative data on any of the administered neuropsychological tests, (iii) imaging quality check was not satisfactory, and (iv) had statistically extreme values on WMLs, based on the Shapiro-Wilk test (*p* > 0.05 [50, 51]) and a visual inspection of the box plots.

### Assessment of purpose in life

PiL was measured through an online self-administered questionnaire using Ryff’s Psychological Well-Being Scale [20]. Within the BBHI framework, this scale was also used to assess personal growth. Additionally, access to further well-being measures was available (please, see [3]). However, in the present study, we only focused on the PiL dimension, as previous investigations have particularly revealed its potential to confer resilience to brain burden (i.e., [34]). In the PiL questionnaire, participants reported from 1 (strongly disagree) to 5 (strongly agree) the following questions: “I live life one day at a time and don’t really think about the future”; “I have a sense of direction and purpose in life”; “I don’t have a good sense of what it is I’m trying to accomplish in life”; “My daily activities often seem trivial and unimportant to me”; “I enjoy making plans for the future and working to make them a reality”; “Some people wander aimlessly through life, but I am not one of them”; and “I sometimes feel as if I’ve done all there is to do in life.” Direct and inverse items were controlled to obtain a total sum score per participant. All included participants answered the complete number of questionnaire items.

### Neuropsychological assessment

Neuropsychological testing was administered by expert neuropsychologists in a single session of approximately 90 min [3, 17]. Tests battery followed a fixed order and included direct and inverse Digit Span [52], Trail Making Test parts A and B [52], Reasoning Matrix [53], Rey Auditory-Verbal Learning Test [54], Block Design Test [53], Letter-Number Sequencing [52], Digit-Symbol Substitution Test and Cancellation subtests from WAIS-IV [53], and Corsi block-tapping test [52].

### MRI acquisition

MRI data were acquired in a 3-T Siemens scanner (MAGNETOM Prisma) with a 32-channel head coil at Unitat d’Imatge per Ressonància Magnètica IDIBAPS (Institut d’Investigacions Biomèdiques August Pi i Sunyer) at Hospital Clínic de Barcelona, Barcelona. For all participants, a high-resolution T1-weighted structural image was obtained with a magnetization-prepared rapid acquisition gradient echo (MPRAGE) three-dimensional protocol (repetition time [TR] = 2400 ms, echo time [TE] = 2.22 ms, inversion time = 1000 ms, field of view [FOV] = 256 mm, flip angle = 8° and 0.8-mm isotropic voxel). Additionally, a high-resolution 3D SPC T2-weighted structural brain MRI was undertaken (TR = 3200 ms, TE = 563 ms, flip angle = 120°, 0.8-mm isotropic voxel, FOV = 256 mm). They also underwent rs-fMRI multiband (anterior-posterior phase-encoding; acceleration factor = 8) interleaved acquisitions (T2-weighted EPI scans, TR = 800 ms, TE = 37 ms, 750 volumes, 72 slices, slice thickness = 2 mm, FOV = 208 mm). All MRI images were examined by a senior neuroradiologist for any clinically significant pathology and visually inspected by trained MRI technicians for subjective quality control of metallic or motion artifacts. To control for movement between rs-fMRI scans, the framewise displacement (FWD) mean was computed.

### Image analyses

The FMRIB Software Library (FSL, version 5.0.11; https://fsl.fmrib.ox.ac.uk/fsl/fslwiki/), Statistical Parametric Mapping (SPM, version 12; https://www.fil.ion.ucl.ac.uk/spm/), and FreeSurfer (version 6.0; https://surfer.nmr.mgh.harvard.edu/) were used for preprocessing and analyzing MRI data. Preprocessing pipeline and head movement considerations are described in SM.

#### White matter burden calculation

Structural T1- and T2-weighted images were automatically processed with FreeSurfer generating white matter hypointensities (WMHs) and estimated total intracranial volume (ETICV) values. Then WMHs were divided by ETICV to obtain an estimate of WMLs adjusted to head volume. This automated method was used as white matter hypointensities and hyperintensities have shown equivalent correlations with age and cerebrospinal fluid (CSF) β-amyloid in non-demented elderly subjects [55]. Moreover, this methodology is strongly associated with other processes used to capture WMLs burden and the Fazekas score [56] and might avoid the potential inclusion of transient lesions [57, 58].

#### Resting-state functional connectivity (rs-FC) analyses

A node-based approach was adopted to quantify individual functional connectivity of resting-state networks (RSNs) as defined in the Shirer atlas of 90 nodes and 14 networks [59]. Blood-oxygen-level-dependent (BOLD) signal was extracted and averaged across all voxels falling within each region of interest (ROI). Then, ROI-to-ROI rs-FCs were computed as Pearson correlations and subsequently Fisher-*Z* transformed. Then, rs-FCs values were included in the calculation of SyS [41], a versatile graph theory-based measure of functional brain network integrity. For this purpose, negative values of rs-FC were set to 0, and autocorrelations were not considered. SyS values of each of the 14 networks were calculated as expressed in:

$${\textrm{SyS}}_{\textrm{net}}=\frac{W_{\textrm{net}}-{B}_{\textrm{net}}}{W_{\textrm{net}}}$$

SyS_net_ captures the balance between within-network (*W*_net_) and between-networks (*B*_net_) rs-FC. *W*_net_ was computed as the average rs-FC connecting all the nodes within the same network, while *B*_net_ was computed as the average rs-FC connecting nodes of a network to nodes from the rest of the brain. Note that two subjects were not considered on the primary visual SyS due to issues during the rs-fMRI processing.

### Statistical analyses

Data analyses were performed using IBM SPSS (IBM Corp. Released 2020. IBM SPSS Statistics, version 27.0. Armonk, NY: IBM Corp) and GraphPad Prism (version 9.0.0, GraphPad Software, San Diego, CA, USA).

First, the total sample was stratified to create two extreme groups, in a similar manner as previous PiL investigations (i.e., [23, 32–34]). With this aim, we used the “visual binning” function from the SPSS, which divides all the included subjects according to a specified number of cut points. The option “equal percentiles based on scanned cases” was used to create 5 sub-groups: Q1 (PiL values: ≤ 21; *N* = 146), Q2 (PiL values: 22–25, *N* = 135), Q3 (PiL values: 26–27, *N* = 114), Q4 (PiL values: 28–30, *N* = 129), and Q5 (PiL values: ≥ 31, *N* = 100). We later conducted analyses focusing on the extreme groups: the Q1, named lower PiL (LP) group, and the Q5, named higher PiL (HP) group. These two groups shaped our sample of interest, conformed by 246 individuals. Basic demographic data (age, gender, YoE) was directly compared between the PiL groups through a one-way analysis of variance (ANOVA) and a chi-squared test. Cognitive data was integrated into three composite scores: an episodic memory composite (EMc), an executive functioning composite (EFc), and a working memory composite (WMc). The EMc was calculated considering the three recall measures from the Rey Auditory-Verbal Learning Test (immediate, delayed, and recognition). The EFc measure was computed considering the Reasoning Matrix, Block Design Test, Digit-Symbol Substitution Test and Cancellation subtests from WAIS-IV. The WMc measure was calculated with the inverse Digit Span and the Letter-Number Sequencing. Composites were obtained through factorial analyses with SPSS. Brain burden was evaluated considering the total estimation of WMLs. rs-fMRI analyses were computed using SyS data on the 14 Shirer circuits [59]. To investigate the neuropsychological differences between the PiL groups, a multivariate general linear model (GLM) was conducted considering all cognitive measures together as dependent variables. Moreover, a univariate GLM with WMLs as the dependent variable was calculated to study the brain burden group differences. Subsequently, Pearson correlations between cognitive status and WMLs in each group, as well as slope differences between the groups, were computed. This latter analysis was conducted with regression functions from GraphPad Prism. In addition, to investigate rs-fMRI differences, a multivariate GLM was undertaken considering the 14 Shirer networks altogether. Whether significant group differences emerged on a whole functional system, a subordinate zoom-in was conducted focused on its ROI-to-ROI functional couplings through multiple GLM analyses, considering within- and between-brain network connectivity. As per its exploratory nature, these analyses were not corrected for multiple comparisons. Finally, Pearson correlations were calculated to relate rs-fMRI measures with cognitive performance in each group. In all the stated statistical analyses, age and gender were used as covariates. YoE were included as a covariate when cognitive data was examined. Moreover, all rs-fMRI explorations were also controlled for FWD. All statistical analyses were two-tailed, and *α* was set at 0.05. For the Pearson correlation analyses, a bootstrapping with 5000 samples was also applied, and the bias-corrected and accelerated 95% confidence interval (CI) was reported. Quality checks were conducted using the stated covariates to corroborate whether the main associations were also present in the whole sample, as well as to further investigate the role of specific variables (i.e., age). Data in plots are presented with standardized *Z* scores, considering the main variables (cognition, WMLs, rs-fMRI) as well as the covariates included in each model (age, gender, YoE, FWD).

## Results

### Neuropsychological and WMLs analyses

**Fig. 1:**
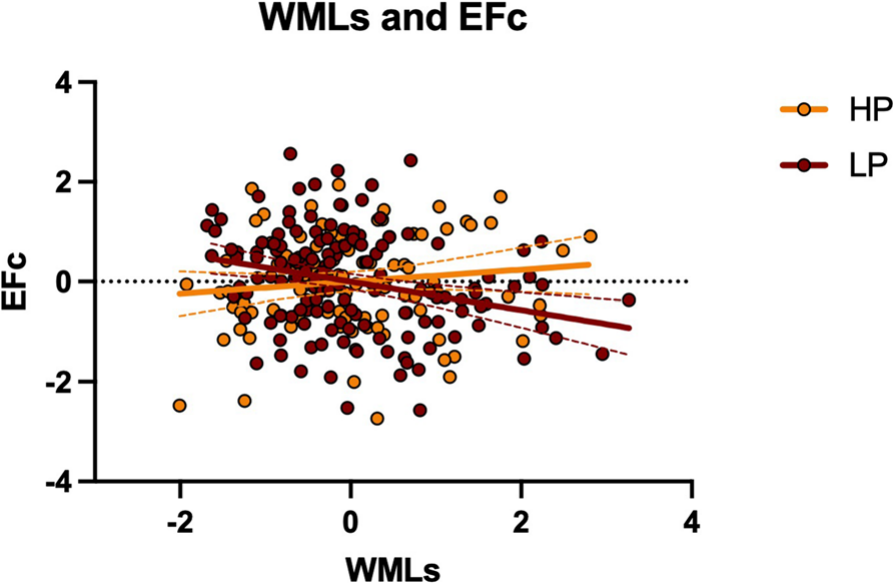
Associations between brain burden and cognition. Scatter plot showing the association between WMLs and executive performance as a function of the PiL group. Data is presented with *Z* scores. *Abbreviations*: EFc, executive functioning composite; HP, higher purpose in life; LP, lower power in life; WMLs, white matter lesions

Neuropsychological status and WMLs did not differ between the HP and LP groups (all *p*-values > 0.05). However, the slopes between the groups (HP vs. LP) were significantly different in the association between EFc and WMLs (*F* = 9.957, *p* = 0.002). Greater WMLs were negatively associated with EFc in the LP group (*r* = − 0.283, *p* < 0.001, 95% CI [− 0.405, − 0.156]) but not in the HP group (*r* = 0.120, *p* = 0.243, 95% CI [− 0.093, 0.324]; Fig. 1). No significant results were observed for the other cognitive measures (all *p*-values > 0.05). As a quality check, in the whole sample, the negative association between EFc and WMLs was also present (*r* = − 0.137, *p* < 0.001, 95% CI [− 0.211, − 0.060]). Moreover, age, which did not differ between the groups (*p* > 0.05; Additional file 1: Table S1), was positively associated with WMLs in both groups (HP group: *r* = 0.345, *p* < 0.001, 95% CI [0.169, 0.499]; LP group: *r* = 0.384, *p* < 0.001, 95% CI [0.223, 0.525]). Demographic data (age, gender, YoE) in each PiL group is further displayed in Additional file 1: Table S1.

### rs-fMRI analyses

**Fig. 2:**
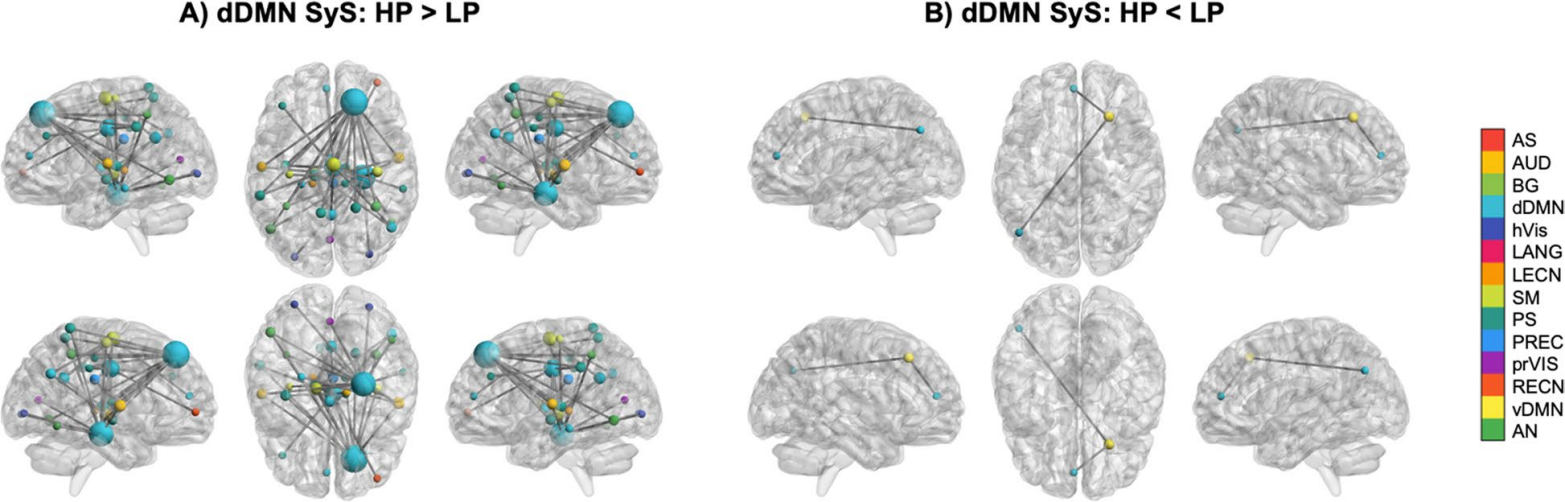
rs-fMRI contrasts between the HP and LP groups. Representation on a standard map of the significant connections between the dDMN nodes and the rest of the brain for **A** HP > LP and **B** HP < LP comparisons. The node size has been generated according to the relative number of edges in each contrast. *Abbreviations*: AN, attentional network; AS, anterior salience network; AUD, auditory network; BG, basal ganglia network; dDMN, dorsal default-mode network; HP, higher purpose in life; hVis, high visual network; LANG, language network; LECN, left executive control network; LP, lower purpose in life; PREC, precuneus network; prVIS, primary visual network; PS, posterior salience network; RECN, right executive control network; SM, sensorimotor network; SyS system segregation; vDMN, ventral default-mode network

Subjects in the HP group showed lower SyS on the dorsal default-mode network than LP subjects (*F* = 4.907, *p* = 0.028), which indicated lesser segregation of this network from other brain circuits. Specifically, HP individuals had greater inter-network connectivity between dDMN nodes and the rest of the brain on the functional couplings depicted in Fig. 2A (see also Additional file 1: Table S2). It is worth noting that, out of the total functional connections identified in this subsequent analysis, three nodes were central: the right superior frontal node, which involved 15 out of 45 of the couplings (33.3%); the hippocampal formation, implicated in 14 out of 45 connections (31.1%), particularly the right hippocampus; and the midcingulate cortex, present in 8 out of 45 functional bridges (17.8%). Altogether, these functional hubs were present in 37 of the 45 functional connections, explaining 82.2% of the results. Conversely, subjects in the LP group exhibited more connectivity than HP individuals across DMN-like regions, particularly those involving connections between the dDMN and the ventral DMN (vDMN; Fig. 2B; Additional file 1: Table S2). The stated DMN-focused ROI-to-ROI analyses comprised a total of 765 comparisons. Of note, no differences were detected in the dDMN between-network connectivity with the other brain circuits nor in the connectivity within the dDMN nodes. Hence, our SyS results were driven by specific dDMN hubs with enhanced inter-connectivity with other brain regions. As a quality check, the negative association between PiL and dDMN SyS was also detected in the whole sample (*r* = − 0.081, *p* = 0.044, 95% CI [− 0.158, − 0.001]).

### Associations between neuropsychological and rs-fMRI data

**Fig. 3:**
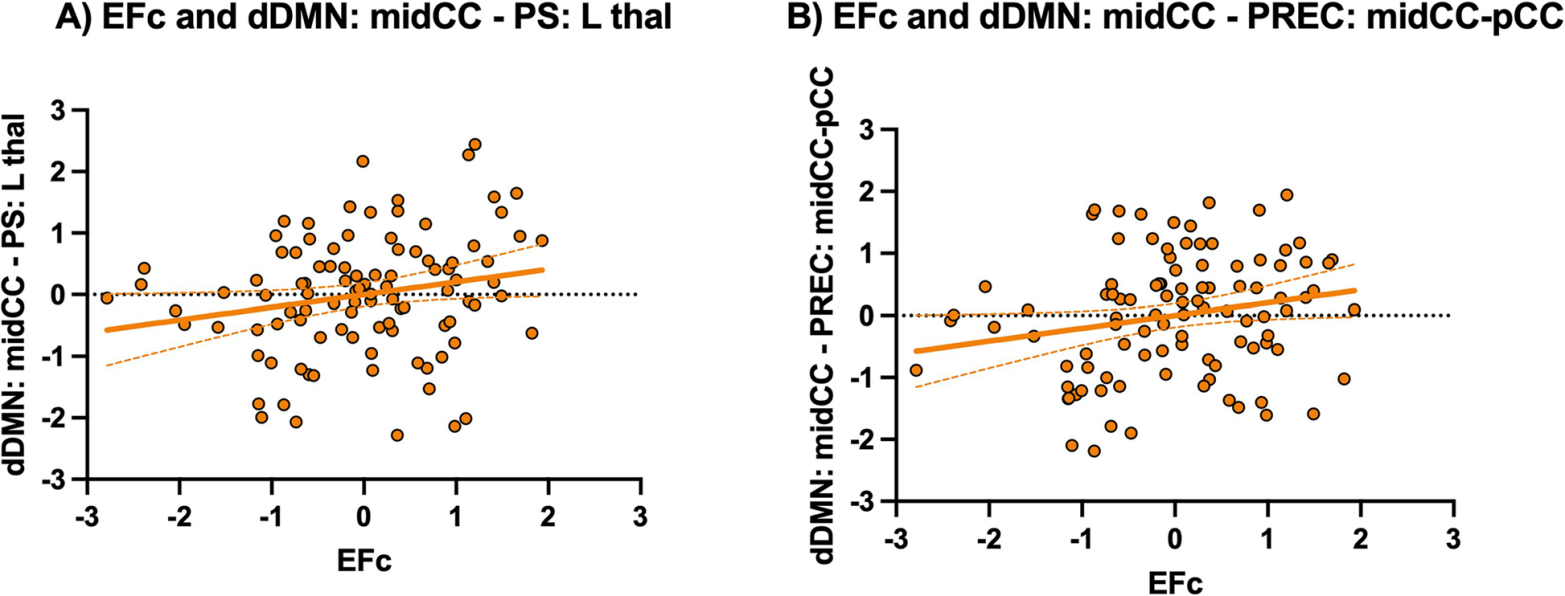
Associations between cognition and rs-FC in the HP group. Scatter plots showing the relationships in the HP group between executive functions and rs-FC between the midcingulate cortex node of the dDMN and **A** the left thalamus node of the posterior salience network and **B** the midcingulate and posterior cingulate node of the precuneus network. Data is presented with *Z* scores. *Abbreviations*: dDMN, dorsal default-mode network; EFc, executive functioning composite; midCC, midcingulate cortex; pCC, posterior cingulate cortex; PREC, precuneus network; PS, posterior salience network; thal, thalamus

Finally, we investigated whether rs-fMRI might differently subtend cognitive function for a given brain burden estimation as a function of the PiL group. Considering the HP > LP identified couplings, we detected two functional connections positively associated with executive performance in the HP group. Both connections involved the midcingulate region of the dDMN and two other cognitive brain systems, the posterior salience (PS), within its thalamic node (*r* = 0.205; *p* = 0.045, 95% CI [0.021, 0.390]; Fig. 3A), and the precuneus network, within its midcingulate and posterior cingulate cortices (*r* = 0.206, *p* = 0.044, 95% CI [0.019, 0.374]; Fig. 3B). No associations with cognition were observed in the LP group (all *p*-values > 0.05). These correlations survived when controlling for WMLs (*r* = 0.204, *p* = 0.047, 95% CI [0.015, 0.378]; *r* = 0.214, *p* = 0.037, 95% CI [0.042, 0.373], respectively). Furthermore, greater values on the variables used in this analysis (EFc and rs-fMRI) were not directly related to WMLs (all *p*-values > 0.05).

## Discussion

In the present study, we first observed that individuals in the highest PiL quintile showed less impact of WMLs on executive functions as compared to those in the lower PiL quintile, indicating greater resilience of HP individuals to brain damage. Subsequently, data revealed that HP participants exhibited lower SyS of the dDMN as compared to LP subjects. At the topographic level, this fact was associated with greater inter-network connectivity between specific dDMN nodes and the rest of the brain, mainly including the frontal cortex, the hippocampal formation, and the midcingulate region. Remarkably, some of these functional couplings supported cognition in the HP group, revealing a possible brain reserve mechanism of PiL among middle-aged adults.

### PiL as a resilience factor in middle-aged individuals

The main result of this investigation was that the association between brain burden and executive performance depends on PiL estimates in middle-aged adults, with subjects with higher PiL exhibiting greater resilience to WMLs. No associations were found regarding episodic or working memory. This aligns with previous literature revealing that WMLs are especially related with executive function and processing speed domains (for a review, see [60]). Furthermore, regarding PiL, previous studies have examined the relationships between this construct and cognitive status, mainly in older samples, with comparable results (i.e., [16, 30, 32]). In a large epidemiologic study of aging, greater sense of purpose was associated with slower rates of cognitive decline and reduced risk of mild cognitive impairment (MCI) and AD [32]. A recent meta-analysis has reinforced these assumptions, revealing that PiL is associated with a reduced risk of dementia [31]. Boyle et al. [34] observed, at the brain level, that superior levels of PiL reduced the deleterious effects of AD pathologic changes on cognitive function. These results suggest that PiL might be a relevant resilience factor, allowing it to counteract the deleterious impact of brain aging and disease on cognitive functions [5, 6]. To the best of our knowledge, this is the first study showing that the PiL-dependent association between brain burden and cognition is also present in healthy middle-aged individuals. Therefore, since according to the current operational definitions (https://reserveandresilience.com/), PiL influenced the associations between a measure of brain burden and cognitive status, it can be considered as a key CR factor, reinforcing the notion that resilience can be influenced by multiple genetic and environmental factors operating continuously across the lifespan [5, 6].

### Functional brain dynamics underlying PiL

The neurobiological mechanisms explaining the pathways by which PiL might promote resilience are poorly understood. Different explanatory models have been proposed to elucidate how PiL relates to cognitive health (i.e., [61]). It is possible that superior PiL estimates are linked to healthier behaviors [27, 28], which are in turn related to better cognitive performance and lower odds of dementia [62]. Alternatively, PiL might relate to biological mechanisms that influence cognitive performance [63]. Thus, scoring higher in PiL has been reported to be associated with lower interleukin-6 (IL-6) plasma concentration levels [64]. Moreover, in middle-aged [65] and older adults [66], greater PiL was found to be related to lower levels of hemoglobin A1c (HbA1c). Also, Ryff’s Psychological Well-Being Scale measures (including PiL) were examined regarding distinct physiological processes (i.e., blood pressure, urinary catecholamines, and salivary cortisol) in a young and middle-aged sample. In this investigation, data revealed that greater PiL is associated with lower total cortisol levels [67]. This is relevant as inflammation, glucose, and cortisol regulation are associated to cognitive and brain health during the lifespan [68–70].

Focusing on brain measures, previous observations have revealed that PiL is linked to a lower risk of cerebrovascular conditions [71, 72], which might confer a resistance role to PiL (i.e., [73]). Furthermore, PiL might help cope with neural damage, providing resilience to brain burden (i.e., [32, 34]). This proposal is consistent with our data, as WMLs were linked to the cognitive status contingent on PiL estimates. Within this context, our study went further on the investigation of the functional brain mechanisms through which PiL may promote cognition, pointing to a particular emphasis on DMN. This aligns with previous investigations that have explored the functional architecture of purpose and sense of life meaning and have revealed a central role of DMN, an archetypical network, which is critically involved in autobiographical remembering, self-referential thought, mental simulation, and mind wandering (i.e., [74–77]). In this sense, Mwilambwe-Tshilobo et al. [78] found that high levels of meaning in life correlated with increased, and more modular, connectivity between the DMN and the limbic system. Additionally, we also identified key regions within the dDMN associated with superior levels of PiL, even though these topographic results should be further clarified. One of them was the hippocampus, a complex brain structure embedded deep into the temporal lobe. This result links with the observations of Waytz et al. [79], reporting that meaning in life is associated to greater connectivity of the medial temporal lobe subsystem of the DMN.

### rs-FC as a mechanism supporting cognition in HP individuals

Overall, while previous findings reinforce the associations between PiL and functional brain measures associated to cognitive function, in our study, we did not found evidence that differences in functional connectivity between the PiL groups, or the associations between HP-related rs-fMRI characteristics and cognitive status, were associated to WMLs burden. These findings therefore indicate that while greater dDMN connectivity among subjects with superior PiL conferred a cognitive advantage on executive functions, this was independent of the deleterious effect of WML in this cognitive domain, and that the brain network’s integrity status was not directly explaining the attenuating effect of high PiL in the relation between WML burden and cognition. Note that these findings differ from those of Ewers et al. [43], where both among familial and sporadic AD, measures of SyS were found to attenuate the effect of pathology (estimated by years to symptom onset in the former and measured by tau-PET in the second) on cognitive function. Within this context, and while our main findings do not challenge the fact that PiL confers resilience to the deleterious effect of WML in middle age, they also suggest that the specific functional mechanism identified here may reflect a brain reserve mechanism (see https://reserveandresilience.com/) but may not involve an active adaptation of functional cognitive processes in the presence of the brain burden measure investigated in the present report.

### PiL as a psychological modifiable factor

While the results of the present study highlight that the brain mechanisms through which high PiL confers resilience in middle age need to be investigated in further research, a relevant aspect is that PiL is a potentially modifiable psychological factor. In this context, psychological interventions, such as meaning-centered psychotherapy (MCP), an extension of classic Frankl’s logotherapy further informed by the contributions of Yalom [80], may be implemented to enhance meaning, spiritual well-being, and quality of life [81–84]. Acceptance and commitment therapy (ACT) might also help individuals to live meaningful lives by encouraging their engagement in activities that are consistent with their values (i.e., [48]). It is relevant to note that improvements in mental health conditions (i.e., anxiety disorders) from psychological therapy have been shown to be associated with reduced incidence of future dementia [85]. Hence, and as the neurobiological underpinnings of both protective and risk psychological factors (i.e., [19, 86]) are being unveiled, such interventional approaches might help to understand how psychological therapies may promote a healthy brain during the lifespan and aid in the prevention of cognitive impairment later in life.

### Limitations

The main limitation of the present study was its cross-sectional nature, which did not allow to explore whether PiL may operate as a resilience mechanism on age-related cognitive decline further than in immediate cognitive status. This also constrains our capacity to infer directionality on the explored variables. Moreover, it is worth highlighting that the use of extreme groups in the present study, as done in previous investigations in the field (i.e., [34]), while allowing the study of PiL concept in a thorough manner, also implies a relevant loss of sample. Finally, part of the neuroimaging results revealing the neurobiological underpinnings of PiL was obtained in an exploratory manner. In particular, the associations between rs-fMRI connectivity measures and cognitive status suggesting a brain reserve effect among PiL needs to be further investigated in forthcoming studies, and in distinct populations (i.e., in the AD continuum).

## Conclusion

The present data extend previous findings found in advanced age and pathological aging, such as AD, revealing that having a strong sense of purpose might confer resilience already in middle age. Furthermore, it was also observed that individuals in the HP group had greater inter-network connectivity between specific dDMN nodes, which correlated with cognitive performance. This may represent a possible brain reserve mechanism related to greater PiL, which needs to be further validated.

## Supplementary Information

**Additional file 1: Table S1.** Demographic data in the higher (HP) and lower purpose in life (LP) groups and differences between them using ANOVAa and chi-squared testsb. Abbreviations: Diff: Differences, M: Men, PiL: Purpose in life, SD: Standard deviation, W: Women, YoE: Years of education. **Table S2.** dDMN functional connections observed when comparing HP vs. LP groups. Data presented has been obtained through GLM analyses. Abbreviations: AN: Attentional network, ang: Angular gyrus, AUD: Auditory network, calcar: Calcarine sulcus, dDMN: Dorsal default-mode network, hipp: Hippocampus, hVis: High visual network, infPar: Inferior parietal sulcus, infTemp: Inferior temporal gyrus, L: Left, LECN: Left executive-control network, medPref-ACC-orb: Medial prefrontal cortex - anterior cingulate cortex - orbitofrontal cortex, midCC-pCC: Midcingulate cortex - posterior cingulate cortex, midCC: Midcingulate cortex, midFront: Middle frontal gyrus, midOcc-supOcc: Middle occipital gyrus, superior occipital gyrus, midTemp: Middle temporal gyrus, postIns-put: Posterior insula – putamen, PREC: Precuneus network, prec: Precuneus, precen-postcen: Precentral gyrus – postcentral gyrus, prVIS: primary visual network, PS: Posterior salience network, R: Right, RECN: Right executive-control network, SM: sensorimotor network, sma: Supplementary motor area, supFront-midFront: Superior frontal gyrus, middle frontal gyrus, supFront: Superior frontal gyrus, supPar-prec: Superior parietal gyrus – precuneus, supramar-infPar: Supramarginal gyrus – inferior parietal gyrus, supTemp-hesc: Superior temporal gyrus - Heschl’s Gyrus, supTemp: Superior temporal gyrus, thal: Thalamus, vDMN: Ventral default-mode network.

## Data Availability

The data that support the findings of this study are available on request from the corresponding authors K.A.-P and D.B.-F.

## Abbreviations

ACT: Acceptance and commitment therapy
AD: Alzheimer’s disease
AN: Attentional network
ANOVA: Analysis of variance
AS: Anterior salience network
AUD: Auditory network
BBHI: Barcelona Brain Health Initiative
BG: Basal ganglia network
Bnet: Between-network
BOLD: Blood-oxygen-level-dependent
CI: Confidence interval
CR: Cognitive reserve
CSF: Cerebrospinal fluid
dDMN: Dorsal default-mode network
EFc: Executive functioning composite
EMc: Episodic memory composite
ETICV: Estimated total intracranial volume
FOV: Field of view
FSL: FMRIB Software Library
FWD: Framewise displacement
GLM: General linear model
HbA1c: Hemoglobin A1c
HP: Higher purpose in life
hVis: High visual network
IL-6: Interleukin-6
LANG: Language network
LECN: Left executive control network
LP: Lower purpose in life
MCI: Mild cognitive impairment
MCP: Meaning-centered psychotherapy
midCC: Midcingulate cortex
MPRAGE: Magnetization prepared rapid acquisition gradient echo
pCC: Posterior cingulate cortex
PiL: Purpose in life
PREC: Precuneus network
prVIS: Primary visual network
PS: Posterior salience network
RECN: Right executive control network
ROI: Region of interest
rs-FC: Resting-state functional connectivity
rs-fMRI: Resting-state functional magnetic resonance imaging
RSNs: Resting-state networks
SM: Sensorimotor network
SPM: Statistical parametric mapping
SyS: System segregation
TE: Echo time
thal: Thalamus
TR: Repetition time
vDMN: Ventral default-mode network
WMc: Working memory composite
WMHs: White matter hypointensities
WMLs: White matter lesions
Wnet: Within-network
YoE: Years of education
