# Supplementary material for: Purpose in life promotes resilience to age-related brain burden in middle-aged adults
Source: Alzheimers Res Ther. 2023 Mar 13;15:49. doi: 10.1186/s13195-023-01198-6 (PMC10009845; doi:10.1186/s13195-023-01198-6)
Supplement: Supplementary file 1 — Additional file 1: Table S1. Demographic data in the higher (HP) and lower purpose in life (LP) groups and differences between them using ANOVAa and chi-squared testsb. Abbreviations: Diff: Differences, M: Men, PiL: Purpose in life, SD: Standard deviation, W: Women, YoE: Years of education. Table S2. dDMN functional connections observed when comparing HP vs. LP groups. Data presented has been obtained through GLM analyses. Abbreviations: AN: Attentional network, ang: Angular gyrus, AUD: Auditory network, calcar: Calcarine sulcus, dDMN: Dorsal default-mode network, hipp: Hippocampus, hVis: High visual network, infPar: Inferior parietal sulcus, infTemp: Inferior temporal gyrus, L: Left, LECN: Left executive-control network, medPref-ACC-orb: Medial prefrontal cortex - anterior cingulate cortex - orbitofrontal cortex, midCC-pCC: Midcingulate cortex - posterior cingulate cortex, midCC: Midcingulate cortex, midFront: Middle frontal gyrus, midOcc-supOcc: Middle occipital gyrus, superior occipital gyrus, midTemp: Middle temporal gyrus, postIns-put: Posterior insula – putamen, PREC: Precuneus network, prec: Precuneus, precen-postcen: Precentral gyrus – postcentral gyrus, prVIS: primary visual network, PS: Posterior salience network, R: Right, RECN: Right executive-control network, SM: sensorimotor network, sma: Supplementary motor area, supFront-midFront: Superior frontal gyrus, middle frontal gyrus, supFront: Superior frontal gyrus, supPar-prec: Superior parietal gyrus – precuneus, supramar-infPar: Supramarginal gyrus – inferior parietal gyrus, supTemp-hesc: Superior temporal gyrus - Heschl’s Gyrus, supTemp: Superior temporal gyrus, thal: Thalamus, vDMN: Ventral default-mode network. [file 13195_2023_1198_MOESM1_ESM.docx]

**Supplementary Material**

**Purpose in life promotes resilience to age-related brain burden in middle-aged adults**

Kilian Abellaneda-Pérez^1,2,3,4,5^, Gabriele Cattaneo^3,4,5^, María Cabello-Toscano^1,2,3^, Javier Solana-Sánchez^3,4,5^, Lídia Mulet-Pons^1,2^, Lídia Vaqué-Alcázar^1,2,6^, Ruben Perellón-Alfonso^1,2^, Cristina Solé-Padullés^1,2^, Núria Bargalló^1,7,8,9^, Josep M. Tormos^3,4,5,10^, Alvaro Pascual-Leone^3,11,12^, David Bartrés-Faz^1,2,3^

^1^Departament de Medicina, Facultat de Medicina i Ciències de la Salut, Institut de Neurociències, Universitat de Barcelona, Barcelona, Spain.

^2^Institut d'Investigacions Biomèdiques August Pi i Sunyer (IDIBAPS), Barcelona, Spain.

^3^Institut Guttmann, Institut Universitari de Neurorehabilitació adscrit a la UAB, Badalona, Barcelona, Spain.

^4^Universitat Autònoma de Barcelona, Bellaterra (Cerdanyola del Vallès), Spain.

^5^Fundació Institut d'Investigació en Ciències de la Salut Germans Trias i Pujol, Badalona, Barcelona, Spain.

^6^Sant Pau Memory Unit, Department of Neurology, Institut d’Investigacions Biomèdiques Sant Pau-Hospital de Sant Pau, Universitat Autònoma de Barcelona, Barcelona, Spain.

^7^Neuroradiology Section, Radiology Department, Diagnostic Image Center, Hospital Clinic of Barcelona, University of Barcelona, Barcelona, Spain.

^8^Magnetic Resonance Image Core Facility (IDIBAPS), Barcelona, Spain.

^9^Centro de Investigación Biomédica en Red de Salud Mental (CIBERSAM), Instituto de Salud Carlos III, Barcelona, Spain.

^10^Centro de Investigación Traslacional San Alberto Magno, Universidad Católica de Valencia San Vicente Mártir, Valencia, Spain.

^11^Hinda and Arthur Marcus Institute for Aging Research and Deanna and Sidney Wolk Center for Memory Health, Hebrew SeniorLife, Boston, MA, USA.

^12^Department of Neurology, Harvard Medical School, Boston, MA, USA.

**Methods**

**Rs-fMRI preprocessing**

The rs-fMRI preprocessing pipeline comprised spatial standardization and nuisance correction by making use of functions from FMRIB Software Library (FSL; version 5.0.11; <https://fsl.fmrib.ox.ac.uk/fsl/fslwiki/>), Statistical Parametric Mapping (SPM, version 12; <https://www.fil.ion.ucl.ac.uk/spm/>) and FreeSurfer (version 6.0; <https://surfer.nmr.mgh.harvard.edu/>). To start with, the first 10 scans were removed to ensure magnetization equilibrium. After that, all images were field inhomogeneity corrected (FSL topup tool), all scans realigned to a reference image (FSL MCFLIRT) and then standardized into native T1-weighted space (SPM Coregister). Finally, normalization (SPM Normalize) of all fMRI images to Montreal Neuroscience Institute (MNI152) standard space was performed to ensure among-subjects comparability. As for nuisance correction, different components were defined and manually removed from the rs-fMRI images by the “fsl_regfilt” tool implemented in FSL. These components correspond to (i) motion regressors of rotation, translation and their derivatives, as estimated during scans’ realignment, (ii) a drift estimated by a discrete cosine transform (DCT) as a low-pass frequency filter (<0.01), and (iii) signals from white matter (WM) and cerebrospinal fluid (CSF). In order to extract these, CSF and WM masks were obtained from automatic subcortical segmentation of brain volume, based upon the existence of an atlas containing probabilistic information on the location of structures [1]. This step was part of the FreeSurfer “recon‐all” processing stream, which was run with default parameters, except for the addition of the T2 flag for the improvement of pial surfaces reconstruction. That is to say, both T1- and T2-weighted images were used for processing anatomical information. Finally, an 8 mm full width at half maximum (FWHM) smoothing filter was applied on the rs-fMRI images.

As head movement may affect rs-fMRI results [2, 3, 4, 5], in-scanner head motion was considered. In this study, the frame-wise displacement (FWD) mean was calculated for every subject. FWD was computed as in Power et al. [2], using the vectors of rotation and translation estimated during scans’ realignment as part of the preprocessing pipeline.

**Results**

**Neuropsychological and WMLs analyses**

|  | **PiL group** | **Mean** | **SD** | **Min** | **Max** | **Group diff** |
| --- | --- | --- | --- | --- | --- | --- |
| **Age** | LP | 53.64 | 7.1 | 42 | 67 | F = 0.816  p = 0.367^a^ |
|  | HP | 54.48 | 7.2 | 42 | 67 |  |
| **Gender** | LP | W = 86  M = 60 | - | - | - | F = 4.609  p = 0.032^b^ |
|  | HP | W = 45  M = 55 | - | - | - |  |
| **YoE** | LP | 16.45 | 3.5 | 8 | 28 | F = 12.071  p < 0.001^a^ |
|  | HP | 18.16 | 4.2 | 8 | 34 |  |

**Table S1.** Demographic data in the higher (HP) and lower purpose in life (LP) groups and differences between them using ANOVA^a^ and chi-squared tests^b^. Abbreviations: Diff: Differences, M: Men, PiL: Purpose in life, SD: Standard deviation, W: Women, YoE: Years of education.

**Rs-fMRI analyses**

| **Functional coupling HP > LP** | **F** | **Sig. (p)** |
| --- | --- | --- |
| AUD: L supTemp-hesc – dDMN: R supFront | 3.871 | 0.050 |
| AUD: L supTemp-hesc – dDMN: R hipp | 5.323 | 0.022 |
| AUD: R supTemp – dDMN: R supFront | 4.653 | 0.032 |
| AUD: R supTemp – dDMN: L hipp | 5.626 | 0.018 |
| AUD: R supTemp – dDMN: R hipp | 8.580 | 0.004 |
| AUD: R thal – dDMN: midCC | 4.107 | 0.044 |
| dDMN: medPref-ACC-orb – SM: R sma | 4.094 | 0.044 |
| dDMN: R supFront – LECN: L thal | 4.917 | 0.028 |
| dDMN: R supFront – SM: R precen-postcen | 4.779 | 0.030 |
| dDMN: R supFront – SM: R sma | 7.703 | 0.006 |
| dDMN: R supFront – SM: L thal | 7.331 | 0.007 |
| dDMN: R supFront – PS: L supramar-infPar | 4.741 | 0.030 |
| dDMN: R supFront – PS: L prec | 4.722 | 0.031 |
| dDMN: R supFront – PS: R midCC | 5.101 | 0.025 |
| dDMN: R supFront – PS: R supPar-prec | 5.518 | 0.020 |
| dDMN: R supFront – PS: L thal | 4.320 | 0.039 |
| dDMN: R supFront – PS: L postIns-put | 4.209 | 0.041 |
| dDMN: R supFront – AN: L infPar | 6.011 | 0.015 |
| dDMN: R supFront – AN: L infTemp | 4.405 | 0.037 |
| dDMN: R supFront – AN: R infPar | 4.849 | 0.029 |
| dDMN: pcc-prec – SM: R sma | 4.152 | 0.043 |
| dDMN: pcc-prec – SM: L thal | 5.508 | 0.020 |
| dDMN: pcc-prec – PS: L thal | 4.038 | 0.046 |
| dDMN: midCC – PS: L midFront | 4.524 | 0.034 |
| dDMN: midCC – PS: L supramar-infPar | 5.031 | 0.026 |
| dDMN: midCC – PS: R supramar-infPar | 4.159 | 0.043 |
| dDMN: midCC – PS: L thal | 4.691 | 0.031 |
| dDMN: midCC – PS: L postIns-put | 4.274 | 0.040 |
| dDMN: midCC – PREC: midCC-pCC | 5.904 | 0.016 |
| dDMN: midCC – RECN: R midFront | 5.332 | 0.022 |
| dDMN: R ang – SM: R sma | 3.929 | 0.049 |
| dDMN: R ang – SM: L thal | 4.452 | 0.036 |
| dDMN: LR thal – PS: R thal | 4.113 | 0.044 |
| dDMN: LR thal – PREC: midCC-pCC | 3.954 | 0.048 |
| dDMN: R hipp – hVIS: L midOcc-supOcc | 5.983 | 0.015 |
| dDMN: R hipp – hVIS: R midOcc-supOcc | 6.614 | 0.011 |
| dDMN: R hipp – SM: L precen-postcen | 4.941 | 0.027 |
| dDMN: R hipp – SM: R precen-postcen | 6.656 | 0.010 |
| dDMN: R hipp – SM: R sma | 6.897 | 0.009 |
| dDMN: R hipp – PS: L prec | 5.205 | 0.023 |
| dDMN: R hipp – PS: R supPar-prec | 4.450 | 0.036 |
| dDMN: R hipp – prVIS: calcar | 4.527 | 0.034 |
| dDMN: R hipp – AN: L infPar | 5.816 | 0.017 |
| dDMN: R hipp – AN: L infTemp | 5.704 | 0.018 |
| dDMN: R hipp – AN: R midTemp | 5.339 | 0.022 |
| **Functional coupling HP < LP** | **F** | **Sig. (p)** |
| dDMN: medPref-ACC-orb – vDMN: R supFront-midFront | 3.938 | 0.048 |
| dDMN: L ang – vDMN: R supFront-midFront | 4.175 | 0.042 |

**Table S2.** dDMN functional connections observed when comparing HP vs. LP groups. Data presented has been obtained through GLM analyses. Abbreviations: AN: Attentional network, ang: Angular gyrus, AUD: Auditory network, calcar: Calcarine sulcus, dDMN: Dorsal default-mode network, hipp: Hippocampus, hVis: High visual network, infPar: Inferior parietal sulcus, infTemp: Inferior temporal gyrus, L: Left, LECN: Left executive-control network, medPref-ACC-orb: Medial prefrontal cortex - anterior cingulate cortex - orbitofrontal cortex, midCC-pCC: Midcingulate cortex - posterior cingulate cortex, midCC: Midcingulate cortex, midFront: Middle frontal gyrus, midOcc-supOcc: Middle occipital gyrus, superior occipital gyrus, midTemp: Middle temporal gyrus, postIns-put: Posterior insula – putamen, PREC: Precuneus network, prec: Precuneus, precen-postcen: Precentral gyrus – postcentral gyrus, prVIS: primary visual network, PS: Posterior salience network, R: Right, RECN: Right executive-control network, SM: sensorimotor network, sma: Supplementary motor area, supFront-midFront: Superior frontal gyrus, middle frontal gyrus, supFront: Superior frontal gyrus, supPar-prec: Superior parietal gyrus – precuneus, supramar-infPar: Supramarginal gyrus – inferior parietal gyrus, supTemp-hesc: Superior temporal gyrus - Heschl’s Gyrus, supTemp: Superior temporal gyrus, thal: Thalamus, vDMN: Ventral default-mode network.

**Bibliography**

1. Fischl B, Salat DH, Busa E, et al. Whole brain segmentation: automated labeling of neuroanatomical structures in the human brain. Neuron. 2002;33(3):341-355. doi:10.1016/s0896-6273(02)00569-x
2. Power JD, Barnes KA, Snyder AZ, Schlaggar BL, Petersen SE. Spurious but systematic correlations in functional connectivity MRI networks arise from subject motion [published correction appears in Neuroimage. 2012 Nov 1;63(2):999]. Neuroimage. 2012;59(3):2142-2154. doi:10.1016/j.neuroimage.2011.10.018
3. Power JD, Mitra A, Laumann TO, Snyder AZ, Schlaggar BL, Petersen SE. Methods to detect, characterize, and remove motion artifact in resting state fMRI. Neuroimage. 2014;84:320-341. doi:10.1016/j.neuroimage.2013.08.048
4. Power JD, Schlaggar BL, Petersen SE. Recent progress and outstanding issues in motion correction in resting state fMRI. Neuroimage. 2015;105:536-551. doi:10.1016/j.neuroimage.2014.10.044
5. Van Dijk KR, Sabuncu MR, Buckner RL. The influence of head motion on intrinsic functional connectivity MRI. Neuroimage. 2012;59(1):431-438. doi:10.1016/j.neuroimage.2011.07.044
